# Protein Adsorption and Conformational Changes

**DOI:** 10.3390/molecules26237079

**Published:** 2021-11-23

**Authors:** Michael Assfalg

**Affiliations:** Department of Biotechnology, University of Verona, 37134 Verona, Italy; michael.assfalg@univr.it

Protein adsorption onto surfaces of diverse materials of both natural and artificial origin is of utmost relevance in many areas of research and technology: medicine, pharmaceutical sciences, analytical sciences, biotechnology, nanotechnology, and cell biology, among others [1,2,3]. In general, any process involving an interface in which contact with a protein solution occurs is likely to be influenced by protein adsorption to the interface [4]. However, despite considerable advances in the field [5,6], our understanding of the mechanisms and consequences of adsorption remains incomplete for a number of reasons. Firstly, the vast diversity of material surfaces makes it impossible to describe, in detail, all the biophysicochemical interactions at play, preventing us from disclosing general paradigms. Secondly, the highly heterogeneous and often dynamic character of interfaces poses tremendous challenges to both experimental and computational approaches, which therefore often provide only partial descriptions. Thirdly, proteins are not uniform, static entities with homogeneous surface properties; instead, they exhibit protein-specific plasticity, complex shapes, and anisotropic surface features.

Proteins adsorb in differing numbers, densities, orientations, and conformations, depending on the chemical and physical properties of the surface and their inherent molecular characteristics [7]. The nature of protein interactions with surfaces ranges from weak Van-der-Waals interactions to strong electrostatic attraction, resulting in a wide range of binding affinities and exchange kinetics. It is well accepted that the properties of surfaces will govern both the degree of conformational change and the orientation of nonspecifically adsorbed proteins [8]. Because of the participation of a significant portion of the protein, adsorption may induce conformational changes from the solution state [9]. Perturbed conformations may result in the impairment of function and exposure of cryptic epitopes that can produce unintended effects in biological signaling [10]. Furthermore, adsorption-induced rearrangements and crowding effects may facilitate protein-protein interactions and aggregation. The latter issue has been intensely investigated, particularly in relation to protein misfolding and amyloid formation. Therefore, molecular-level descriptions of protein adsorption would significantly contribute to our understanding of the phenomenon [11], and are essential for the effective control of protein–surface interactions.

To rationalize the way in which proteins interact with surfaces, it is useful to consider the peculiarities of the different types of surfaces. For example, it is known that nanomaterials exhibit unique properties that differ from those of the bulk substances, and are therefore attracting considerable interest in many areas of medicine and technology. As opposed to planar surfaces, nanoparticles have a very large surface-to-volume ratio, so that even small amounts of particles present extremely large surface areas available for protein binding. Moreover, nanoparticle surface curvature has a profound influence on the adsorption process. Thus, protein-nanoparticle interactions have been extensively investigated, and it is not surprising that they are discussed in a number of reports in this Special Issue [12,13,14,15]. Lipid surfaces are instead the subject of another contribution in this issue [16]. Lipid layers are indeed important mimics of biological membranes and may be employed to assemble solid-supported membranes, which are well-suited for studying conformational dynamics phenomena of peripheral or integral membrane proteins.

In this Issue, Perera et al. [12] describe a systematic study of peptide and protein adsorption to PEGylated gold nanoparticles. PEGylation is a universally adopted strategy for the passivation of nanoparticles, which reduces unintended protein adsorption and extends the lifetime of gold nanoparticles in biofluids. Systematic investigations are crucial to establish quantitative relationships between molecular/particle properties and binding propensities. The authors use an NMR spectroscopy-based approach, which allows them to obtain the in situ quantification of protein binding and the determination of association constants and kinetic parameters. The study reveals important determinants of the differing penetration of test proteins into PEG coatings of variable density and thickness.

Protein interaction with gold nanoparticles is also the subject discussed in another original report of this issue, contributed by Hunashal et al. [13], although in this case, the focus is on the influence of nanoparticles on protein fibrillogenesis. The authors introduce a method of investigation of site-specific transient interactions based on NMR signal perturbations induced by an extrinsic paramagnetic agent. Their study involves citrate-stabilized gold nanoparticles and β2-microglobulin, a paradigmatic example of amyloidogenic protein. Paramagnetic perturbation mapping provides insight into protein regions which become incompetent for protein–protein interactions in the presence of nanoparticles, thereby contributing to explain why these nanoobjects inhibit the fibrillogenesis of this protein.

Alpha-synuclein is another prototypical amyloidogenic protein, associated with a number of neurodegenerative conditions, including Parkinson’s disease. Different from β2-microglobulin, alpha-synuclein is intrinsically disordered in solution, but undergoes conformational rearrangement in pathological conditions, or upon proper stimulation in vitro. The review by D’Onofrio et al. [15] summarizes a large number of studies that have been conducted to understand how the unstructured polypeptide adapts to nanoparticle surfaces, and how its adsorption influences protein self-assembly and formation of fibrillar structures. Indeed, nanoparticles represent an attractive alternative to traditional drugs for the development of agents capable of mitigating aberrant protein aggregation in devastating brain diseases. The conformational plasticity of alpha-synuclein further offers interesting opportunities for the development of new nanobiomaterials displaying emergent properties.

Georgieva reviewed the current progress of research based on the use of electron paramagnetic resonance (EPR) to study the protein conformational dynamics of proteins associated with surfaces of membranes or nanomaterials [14]. The author describes how EPR in its continuous-wave and pulse spectroscopy modalities, in combination with spin labeling, provides information on local dynamics and long-range conformational rearrangements of proteins. The reported case studies include investigations of the disordered, amyloidogenic proteins tau and alpha-synuclein on interaction with lipid membranes, which contributed significant insights into the structural changes experienced by the proteins a upon transition from the solution to the lipid layer. The review further considers examples of application of the technique to investigate the conformational behavior of proteins immobilized through adsorption onto surfaces of engineered nanomaterial particles and synthetic membranes, at the basis of new technological developments in biotechnology and analytical sciences.

Protein immobilization on engineered membranes is indeed a convenient preparative procedure for investigations of membrane protein function. Tadini-Buoninsegni reviewed research articles focused on applications of an electrophysiological method based on solid supported membranes (SSMs) to elucidate the ion transport mechanism in P-type ATPases, a superfamily of membrane transporters. During the catalytic cycle, the ATPases typically undergo structural rearrangements and conformational transitions to perform ion transport across the membrane. Membrane layers or vesicles that embed the transport protein are adsorbed on the SSM. The proteins are activated by a substrate concentration jump at the SSM, and the charge translocations during the reaction cycle are monitored by measuring a transient current signal. The described approach has a broad scope, and also finds application in drug discovery, due to its ability to monitor protein/drug interactions. One could envision that the study of protein/nanoparticle interactions on SSMs could be another field of interest in the future.

In summary, protein adsorption to surfaces plays a crucial role in both basic and applied research. The reports contained in this Special Issue show that significant progress has been made in our understanding of the adsorption mechanisms, and of complex conformational dynamics at interfaces, contributing to set the basis for future developments.

I wish to thank the authors for their contributions to this Special Issue, all the reviewers for their invaluable work, and the editorial staff of *Molecules*, particularly Assistant Editor Emity Wang for her kind assistance during the preparation of this Special Issue.
